# Association of Sociodemographic Characteristics With 1-Year Hospital Readmission Among Adults Aged 18 to 55 Years With Acute Myocardial Infarction

**DOI:** 10.1001/jamanetworkopen.2022.55843

**Published:** 2023-02-14

**Authors:** Chinenye M. Okafor, Cenjing Zhu, Valeria Raparelli, Terrence E. Murphy, Andrew Arakaki, Gail D’Onofrio, Sui W. Tsang, Marcella Nunez Smith, Judith H. Lichtman, John A. Spertus, Louise Pilote, Rachel P. Dreyer

**Affiliations:** 1 Department of Chronic Disease Epidemiology, Yale School of Public Health, New Haven, Connecticut; 2 Department of Translational Medicine, University of Ferrara, Ferrara, Italy; 3 University Center for Studies on Gender Medicine, University of Ferrara, Ferrara, Italy; 4 Faculty of Nursing, University of Alberta, Edmonton, Alberta, Canada; 5 Department of Public Health Sciences, Pennsylvania State University College of Medicine, Hershey; 6 Department of Emergency Medicine, Yale School of Medicine, New Haven, Connecticut; 7 Program on Aging, Department of Internal Medicine, Yale School of Medicine, New Haven, Connecticut; 8 Equity Research and Innovation Center, Yale School of Medicine, New Haven, Connecticut; 9 School of Medicine, University of Missouri, Kansas City; 10 Department of Cardiovascular Research, Saint Luke’s Mid America Heart Institute, Kansas City, Missouri; 11 Division of Clinical Epidemiology, McGill University Health Centre, Montreal, Quebec, Canada; 12 Division of General Internal Medicine, McGill University Health Centre, Montreal, Quebec, Canada; 13 Center for Outcomes Research and Evaluation, McGill University Health Centre Research Institute, Montreal, Quebec, Canada; 14 Department of Biostatistics, Yale School of Public Health, New Haven, Connecticut

## Abstract

**Importance:**

Among younger adults, the association between Black race and postdischarge readmission after hospitalization for acute myocardial infarction (AMI) is insufficiently described.

**Objectives:**

To examine whether racial differences exist in all-cause 1-year hospital readmission among younger adults hospitalized for AMI and whether that difference retains significance after adjustment for cardiac factors and social determinants of health (SDOHs).

**Design, Setting, and Participants:**

The VIRGO (Variation in Recovery: Role of Gender on Outcomes of Young AMI Patients) study was an observational cohort study of younger adults (aged 18-55 years) hospitalized for AMI with a 2:1 female-to-male ratio across 103 US hospitals from January 1, 2008, to December 31, 2012. Data analysis was performed from August 1 to December 31, 2021.

**Main Outcomes and Measures:**

The primary outcome was all-cause readmission, defined as any hospital or observation stay greater than 24 hours within 1 year of discharge, identified through medical record abstraction and clinician adjudication. Logistic regression with sequential adjustment evaluated racial differences and potential moderation by sex and SDOHs. The Blinder-Oaxaca decomposition quantified how much of any racial difference was explained and not explained by covariates.

**Results:**

This study included 2822 participants (median [IQR] age, 48 [44-52] years; 1910 [67.7%] female; 2289 [81.1%] White and 533 [18.9%] Black; 868 [30.8%] readmitted). Black individuals had a higher rate of readmission than White individuals (210 [39.4%] vs 658 [28.8%], *P* < .001), particularly Black women (179 of 425 [42.1%]). After adjustment for sociodemographic characteristics, cardiac factors, and SDOHs, the odds of readmission were 34% higher among Black individuals (odds ratio [OR], 1.34; 95% CI, 1.06-1.68). The association between Black race and 1-year readmission was positively moderated by unemployment (OR, 1.68; 95% CI, 1.09- 2.59; *P* for interaction = .02) and fewer number of working hours per week (OR, 1.01; 95% CI, 1.00-1.02; *P* for interaction = .01) but not by sex. Decomposition indicates that 79% of the racial difference in risk of readmission went unexplained by the included covariates.

**Conclusions and Relevance:**

In this multicenter study of younger adults hospitalized for AMI, Black individuals were more often readmitted in the year following discharge than White individuals. Although interventions to address SDOHs and employment may help decrease racial differences in 1-year readmission, more study is needed on the 79% of the racial difference not explained by the included covariates.

## Key Points

**Question:**

Are race and social determinants of health associated with hospital readmission within 1 year of discharge for acute myocardial infarction?

**Findings:**

Among 2822 younger adults, Black individuals had an adjusted 34% higher odds of readmission vs White individuals, with Black women having the highest prevalence. This association of Black race with readmission was stronger among unemployed individuals and those with fewer hours of work.

**Meaning:**

Black individuals, especially women, appear to have an excess risk of 1-year readmission not explained by a higher burden of risk factors or social determinants of health, so future research should identify factors associated with readmission that may be addressed to improve equity.

## Teaser

This cohort study of patients with acute myocardial infarction (AMI) assesses whether race and social determinants of health are associated with hospital readmission rates within 1 year of discharge.

## Introduction

Cardiovascular disease remains the leading cause of death in the US,^1^ disproportionately affecting racial minority groups,^2^ such as Black individuals.^1,2^ Specific to coronary heart disease, studies have demonstrated that Black individuals of all ages, when compared with White individuals, have a higher risk of mortality^3^ and hospital readmission up to 1 year after acute myocardial infarction (AMI).^4,5,6,7^ Younger Black adults (aged <55 years) with AMI, especially women, are a particularly high-risk group because they have been shown to receive lower in-hospital quality of care^8,9^ and have higher rates of 1-year cardiac readmission and mortality.^9^ Despite this disparity and the urgent need to reduce AMI readmission among younger individuals, factors contributing to the elevated risk of adverse outcomes among racial and ethnic minority groups with AMI remain understudied.

Social determinants of health (SDOHs), including social support, socioeconomic status, and residential environment, have been explored extensively with regard to their association with cardiovascular health and outcomes.^2,10,11^ Furthermore, psychosociocultural factors reportedly exert a greater impact on the risk of 30-day readmission among younger vs older individuals with AMI.^12^ Although studies have reported increasing hospitalization burden beyond the typical 30-day readmission period after AMI,^12,13^ little is known about how race and SDOHs affect long-term readmission, especially among younger AMI survivors. Moreover, factors that may mediate the association between race and readmission at 1 year after AMI in younger Black individuals, such as SDOHs or biological sex, remain largely unexplored.

To address this gap in knowledge, our objectives were to (1) examine racial differences in the rate of all-cause hospital readmission 1 year after AMI; (2) assess the association of race with 1-year readmission by sequential adjustment for cardiac risk factors, comorbidities and medical history, disease severity, and SDOHs; and (3) examine whether these factors are more strongly associated with Black vs White race. Our hypothesis was that Black younger adults have higher rates of all-cause readmission compared with their White counterparts and that the independent association of SDOHs and biologic sex with 1 year of readmission would be stronger in Black than White individuals.

## Methods

### Study Participants and Design

We carried out analyses using data from the VIRGO (Variation in Recovery: Role of Gender on Outcomes of Young AMI Patients) study from August 1 to December 31, 2021. Details of the VIRGO study have been previously described.^14^ In brief, VIRGO was a multicenter, prospective cohort study of younger adults aged 18 to 55 years who had an AMI between August 1, 2008, and May 31, 2012, and were hospitalized at 1 of 103 hospitals in the US (94 of 103 [91.3%] were academic hospitals; median bed size, 536). Patients were recruited using a 2:1 female-to-male ratio. The study was designed to examine the differences in the distribution and prognostic importance of demographic, clinical, psychosocial, behavioral, and biological risk factors associated with worse outcomes among younger adults with AMI. For the current analysis, participants from the US who self-reported their race as Black or White were included and those who self-reported being American Indian or Alaska Native (n = 33); Asian, Pacific Islander, or East Indian (n = 70); Hispanic (n = 38); or unknown race (n = 21) were excluded. Institutional review board approval was obtained at each participating institution, and patients provided written informed consent for their study participation, including baseline hospitalization and follow-up interviews. This study followed the Strengthening the Reporting of Observational Studies in Epidemiology (STROBE) reporting guidelines for cohort studies.

### Baseline Characteristics and SDOHs

Trained personnel abstracted data from medical records and conducted standardized in-person interviews during the index AMI admission. Variables were classified into categories of demographic factors, cardiac risk factors, comorbidities and medical history, presentation and disease severity, and SDOHs.

We included variables on demographic factors, such as age, biological sex (self-reported during screening phase), and self-reported race, and cardiac risk factors including diabetes, obesity (body mass index ≥30 [calculated as weight in kilograms divided by height in meters squared]), hypertension, dyslipidemia, current smoking, family history of cardiovascular disease, and physical inactivity. Physical activity was self-reported and measured using the Behavioral Risk Factor Surveillance Survey physical activity instrument, which has been shown to be a reliable and valid measure among younger adults.^15^ Comorbidities included history of kidney disease, alcohol abuse, chronic obstructive pulmonary disease, stroke, peripheral artery disease, drug use, and depression. The Patient Health Questionnaire 9 was used to measure depressive symptoms,^16^ with higher scores representing higher levels of depression. Severity of participants’ coronary AMI was quantified by prior AMI, ejection fraction greater than 40%, heart failure, AMI type (non–ST-segment elevation myocardial infarction or ST-segment elevation myocardial infarction), and admission to the cardiac or medical intensive care unit.

The SDOH variables were included according to the World Health Organization definition and conceptual framework of SDOHs.^17,18,19^ This broader definition of SDOHs has been used in prior studies.^20,21^ These variables included low socioeconomic status, employment status, number of work hours per week, marital status, household primary earner status, burden of stress, support for household chores, social support, and health insurance. Low socioeconomic status was defined using a combination of educational level (less than high school) or the 2 lowest categories of personal income (≤$30 000). Social support was quantified with the 7-item Enhancing recovery in Coronary Heart Disease Patients Social Support instrument (ESSI).^22,23^ Consistent with the approach used in other studies,^3,21,24,25^ we excluded instrumental support or household chores and marital status to produce the composite 5-item ESSI. Higher scores on the 5-item ESSI represented greater perceived social support. Low social support was then defined as a score of 3 or less on at least 2 items of the ESSI and a total ESSI score of 18 or less.^9,26^

### Primary Outcome and Adjudication of 1-Year All-Cause Readmission

The primary outcome was all-cause readmission, defined as any hospital or observation stay greater than 24 hours within 1 year of discharge. The readmission adjudication process has been described elsewhere.^25^ In brief, during the 1-year follow-up period, research coordinators at each VIRGO site identified all-cause readmissions through medical record abstraction and participants’ self-report of any readmission (with records obtained from other hospitals), which was then collated and adjudicated by the Yale Coordinating Center. Following extensive training using a custom-developed Research Electronic Data Capture (REDCap) external module, 5 physicians and an advanced practice registered nurse at Yale University completed adjudication for each readmission.

### Statistical Analysis

Missingness in both outcomes and explanatory variables was low (<5% in the study cohort and for each category of 1-year readmission) (eTable 1 in Supplement 1). We consequently assumed missing at random and conducted complete case analyses. We summarized baseline characteristics for the total population and compared outcomes and explanatory variables between Black and White races within strata based on readmission status. The Kruskal-Wallis and χ^2^ tests were used for continuous and categorical variables, respectively.

Unadjusted logistic models were used to examine the associations of demographic characteristics, cardiac risk factors, comorbidities and medical history, presentation characteristics, disease severity, and SDOHs with the likelihood of all-cause readmission at 1 year. The adjusted association of race with 1-year all-cause readmission after AMI was determined using a multivariable logistic regression model that sequentially adjusted for prespecified domains of covariates selected based on statistical significance and clinical judgment.^9,25^

Model 1 included age, self-reported race, and sex. Model 2 included self-reported race, age, sex, hypertension, diabetes, obesity, dyslipidemia, current smoking, physical activity, prior AMI, history of kidney disease, history of depression, and AMI type. Model 3 further included any additional SDOHs that were statistically significant in univariate analyses. Results from multivariable analyses are presented as odds ratios (ORs) with 95% CIs. Using the Wald χ^2^ test, 2-way interactions were serially tested between race and any SDOH significant in univariate analyses and with sex in all 3 models. The Blinder-Oaxaca 2-way decomposition evaluated what proportions of any racial difference were explained and not explained by the covariates that remained significant in model 3. Fully adjusted models were also separately fit to each racial subgroup, with results reported in eTable 2 in Supplement 1. Finally, we conducted sensitivity analyses accounting for site-specific fixed effects by including least-squared dummy variables in a multivariable model using R software, version 1.4 (R Foundation for Statistical Computing) and performed the Blinder-Oaxaca decomposition with Stata software, version 16.1 (StataCorp LLC). All other statistical analyses were performed using SAS software, version 9.4 (SAS Institute Inc), with 2-tailed tests for statistical significance at α = .05.

## Results

### Baseline Characteristics

This study included 2822 participants (median [IQR] age, 48 [44-52] years; 1910 [67.7%] female; 2289 [81.1%] White and 533 [18.9%] Black). Black individuals had a higher prevalence of diabetes, obesity, hypertension, family history of cardiovascular disease, and a sedentary lifestyle at baseline when compared with their White counterparts (Table 1). They were also more likely to have prior AMI, stroke, kidney disease, and heart failure at baseline hospitalization. A total of 868 participants (30.8%) were readmitted during the first year after an AMI. Black individuals were readmitted more often compared with their White counterparts (210 [39.4%] vs 658 [28.8%]; *P* < .001). In the comparison of readmission by race and sex, the highest readmission rate among the 4 groups (ie, Black women, Black men, White women, and White men) was observed among Black women (179 of 425 [42.1%]).

**Table 1.** Baseline Characteristics and Social Determinants of Health of Younger Black and White Adults Hospitalized for Acute Myocardial Infarction in Strata Based on Readmission Status^a^

Characteristic	Total (N = 2822)	Readmitted (n = 868)		*P* value^b^	Not readmitted (n = 1954)		*P* value^b^
		Black (n = 210)	White (n = 658)		Black (n = 323)	White (n = 1631)	
Demographic characteristics							
Age, median (IQR), y	48.0 (44.0-52.0)	47.0 (41.0-51.0)	48.0 (44.0-52.0)	.02	47.0 (44.0-53.0)	49.0 (44.0-52.0)	.005
Sex							
Female	1910 (67.7)	179 (85.2)	476 (72.3)	<.001	246 (76.2)	1009 (61.9)	<.001
Male	912 (32.3)	31 (14.8)	182 (27.7)		77 (23.8)	622 (38.1)	
Cardiac risk factors							
Diabetes	1003 (35.5)	115 (54.8)	275 (41.8)	.001	118 (36.5)	495 (30.4)	.03
Obesity (BMI≥30)	1511 (53.6)	137 (65.2)	351 (53.3)	.003	196 (60.7)	827 (50.8)	.001
Hypertension	1872 (66.3)	185 (88.1)	445 (67.6)	<.001	253 (78.3)	989 (60.6)	<.001
Dyslipidemia	2444 (86.6)	187 (89.1)	580 (88.2)	.72	263 (81.2)	1414 (86.7)	.01
Current smoking	813 (28.8)	65 (31.0)	174 (26.4)	.20	100 (31.0)	474 (29.1)	.49
Family history of CVD	1908 (67.8)	127 (60.5)	478 (72.6)	<.001	189 (58.7)	1114 (68.6)	<.001
Inactivity	996 (35.3)	109 (51.9)	252 (38.3)	<.001	141 (43.7)	494 (30.3)	<.001
Comorbidities and medical history							
History of kidney disease	322 (11.5)	43 (20.7)	84 (12.8)	.005	30 (9.3)	165 (10.2)	.65
Alcohol abuse	978 (34.7)	52 (24.8)	211 (32.1)	.04	107 (33.1)	608 (37.3)	.16
History							
COPD	329 (11.7)	33 (15.7)	109 (16.6)	.77	27 (8.4)	160 (9.8)	.42
Stroke	96 (3.4)	21 (10.0)	22 (3.4)	<.001	18 (5.6)	35 (2.2)	<.001
PAD	69 (2.5)	8 (3.8)	31 (4.7)	.58	6 (1.9)	24 (1.5)	.60
Recreational drug use	44 (1.6)	7 (3.3)	13 (2.0)	.25	6 (1.9)	18 (1.1)	.26
Depression	1176 (41.7)	78 (37.1)	359 (54.6)	<.001	86 (26.6)	653 (40.1)	<.001
Presentation characteristics and disease severity							
Prior MI	609 (21.6)	77 (36.7)	173 (26.3)	.004	75 (23.2)	284 (17.4)	.01
History of heart failure	131 (4.6)	40 (19.1)	35 (5.3)	<.001	27 (8.4)	29 (1.8)	<.001
Ejection fraction >40%	300 (11.0)	31 (15.4)	75 (11.8)	.18	34 (10.9)	160 (10.1)	.68
Type of MI							
STEMI	1408 (49.9)	84 (40.0)	315 (47.9)	.04	128 (39.6)	881 (54.0)	<.001
NSTEMI	1414 (50.1)	126 (60.0)	343 (52.1)		195 (60.4)	750 (46.0)	
Admitted to CCU or ICU	119 (4.2)	8 (3.8)	31 (4.7)	.58	20 (6.2)	60 (3.7)	.04
Social determinants of health							
Low SES	1203 (44.1)	131 (65.5)	321 (50.0)	<.001	185 (60.1)	566 (35.8)	<.001
Currently unemployed	1097 (38.9)	130 (61.9)	298 (45.3)	<.001	136 (42.1)	533 (32.7)	.001
No. of work hours per week, median (IQR)	32.0 (0-40.0)	0 (0-39.0)	20.0 (0-40.0)	<.001	35.5 (0-40.0)	38.0 (0-43.0)	<.001
Married or living with spouse	1562 (53.4)	62 (29.5)	367 (55.8)	<.001	124 (38.4)	1009 (61.9)	<.001
Primary earner	2097 (74.3)	134 (63.8)	475 (72.2)	.02	223 (69.0)	1265 (77.6)	.001
High burden of stress	1391 (49.8)	102 (50.5)	381 (58.4)	.047	134 (42.0)	774 (47.8)	.06
Support for household chores	1801 (64.6)	109 (53.7)	422 (64.9)	.004	203 (64.2)	1067 (65.8)	.59
Low social support	592 (21.3)	55 (27.4)	147 (22.7)	.18	69 (21.9)	321 (19.9)	.41
Has health insurance	2183 (77.6)	159 (75.7)	520 (79.3)	.28	233 (72.1)	1271 (78.2)	.02

Abbreviations: BMI, body mass index (calculated as weight in kilograms divided by height in meters squared); CCU, critical care unit; COPD, chronic obstructive pulmonary disease; CVD, cardiovascular disease; ICU, intensive care unit; MI, myocardial infarction; NSTEMI, non–ST-segment elevation myocardial infarction; PAD, peripheral artery disease; SES, socioeconomic status; STEMI, ST-segment elevation myocardial infarction.

^a^ Data are presented as number (percentage) of patients unless otherwise indicated.

^b^ *P* values for comparison of Black and White race within strata defined by readmission status from Kruskal-Wallis and χ^2^ tests for continuous and categorical variables, respectively.

### SDOHs and 1-Year All-Cause Readmission by Race

All SDOHs except low social support and having health insurance were significantly different across 1-year readmission status and racial groups (Table 1). Compared with their White counterparts, Black individuals who were readmitted had higher rates of unfavorable metrics on most of the measured SDOHs. Specifically, Black compared with White individuals who were readmitted had higher rates of social metrics such as low socioeconomic status (131 [65.5%] vs 321 [50.0%]), unemployment (130 [61.9%] vs 298 [45.3%]), and fewer median (IQR) work hours per week (0 [0-39.0] vs 20.0 [0-40.0]). They also reported lower rates of metrics measured as married or living with spouse (62 [29.5%] vs 367 [55.8%]), primary earner (134 [63.8%] vs 475 [72.2%]), and having support for household chores (109 [53.7%] vs 422 [64.9%]) relative to White individuals with similar readmission status at 1 year. Of interest, readmitted White individuals reported a higher burden of baseline stress vs readmitted Black individuals (381 [58.4%] vs 102 [50.5%]).

### Factors Associated With 1-Year All-Cause Readmission

Univariate analysis showed that Black participants had 61% greater odds (OR, 1.61; 95% CI, 1.32-1.96) of being readmitted at 1 year compared with White participants (eTable 3 in Supplement 1). Female sex was also associated with greater odds of being readmitted at 1 year (OR, 1.71; 95% CI, 1.43-2.05), but this association did not differ by race. Furthermore, cardiac risk factors (diabetes, hypertension, and physical inactivity) and comorbidities, including prior AMI and history of depression, were associated with 1-year readmission. Participants who experienced non–ST-segment elevation myocardial infarction had 26% greater odds of being readmitted at 1 year compared with participants who had an ST-segment elevation myocardial infarction. Among the SDOHs considered, participants with low socioeconomic status, low social support, and high burden of stress had greater odds of 1-year readmission. Participants who were unemployed at baseline had 87% (OR, 1.87; 95% CI, 1.59-2.20) higher odds of readmission at 1 year.

The results of the final adjusted multivariable model for readmission at 1 year are given in Table 2, and the unadjusted and adjusted associations between race and readmission are presented in the Figure. In model 1, Black race was associated with all-cause 1-year readmission (OR, 1.49; 95% CI, 1.22-1.82), and this association remained significant after adjustment for cardiac risk factors, comorbidities, and disease severity in model 2 (OR, 1.38; 95% CI, 1.11-1.70) and adjustment for SDOHs in model 3 (OR, 1.34; 95% CI, 1.06-1.68). Sensitivity analyses accounting for the clustering of participants within sites demonstrated similar results (eTable 4 in Supplement 1). All SDOHs with significant unadjusted associations were separately tested for their interactions with race; the interactions between race and current unemployment (OR, 1.68; 95% CI, 1.09-2.59; *P* for interaction = 0.02) and the interaction between race and incremental reduction in work hours per week (OR, 1.01; 95% CI, 1.00-1.02; *P* for interaction = 0.01) were significant.

**Table 2.** Multivariable Model of Factors Associated With 1-Year All-Cause Readmission in Younger Adults With AMI

Variable	OR (95% CI)		
	Model 1	Model 2	Model 3
Demographic characteristics			
Self-reported Black race	1.49 (1.22-1.82)^a^	1.38 (1.11-1.70)^b^	1.34 (1.06-1.68)^c^
Female sex	1.64 (1.37-1.97)^a^	1.44 (1.19-1.75)^b^	1.29 (1.05-1.59)^c^
Age	0.99 (0.98-1.00)	0.98 (0.97-0.99)^b^	0.98 (0.97-1.00)^c^
Cardiac risk factors			
Diabetes	NA	1.45 (1.21-1.74)^b^	1.46 (1.21-1.76)^c^
Hypertension	NA	1.14 (0.94-1.39)	1.09 (0.89-1.34)
Obesity	NA	0.91 (0.77-1.09)	0.90 (0.75-1.08)
Dyslipidemia	NA	1.10 (0.85-1.43)	1.07 (0.82-1.41)
Current smoking	NA	0.96 (0.80-1.16)	1.06 (0.87-1.30)
Inactivity	NA	1.29 (1.08-1.53)^b^	1.25 (1.04-1.49)^c^
Comorbidities and medical history			
Prior MI	NA	1.53 (1.25-1.87)^b^	1.43 (1.16-1.76)^c^
History			
Kidney disease	NA	1.32 (1.03-1.70)^b^	1.29 (0.99-1.68)
Depression	NA	1.45 (1.22-1.72)^b^	1.33 (1.10-1.60)^c^
			
AMI type	NA	1.10 (0.93-1.30)	1.07 (0.90-1.28)
SDOHs			
Low SES	NA	NA	1.13 (0.92-1.40)
Currently unemployed	NA	NA	1.07 (0.72-1.59)
Incremental reduction in work hours per week	NA	NA	1.00 (1.00-1.01)
Married or living with spouse	NA	NA	1.03 (0.83-1.26)
Primary earner	NA	NA	0.84 (0.67-1.05)
High burden of stress	NA	NA	1.24 (1.04-1.49)^c^
Low social support	NA	NA	1.04 (0.84-1.28)

Abbreviations: AMI, acute myocardial infarction; MI, myocardial infarction; NA, not applicable; OR, odds ratio; SDOHs, social determinants of health; SES, socioeconomic status.

^a^ Statistically significant in first model.

^b^ Statistically significant in second model.

^c^ Statistically significant in final model.

**Figure.**
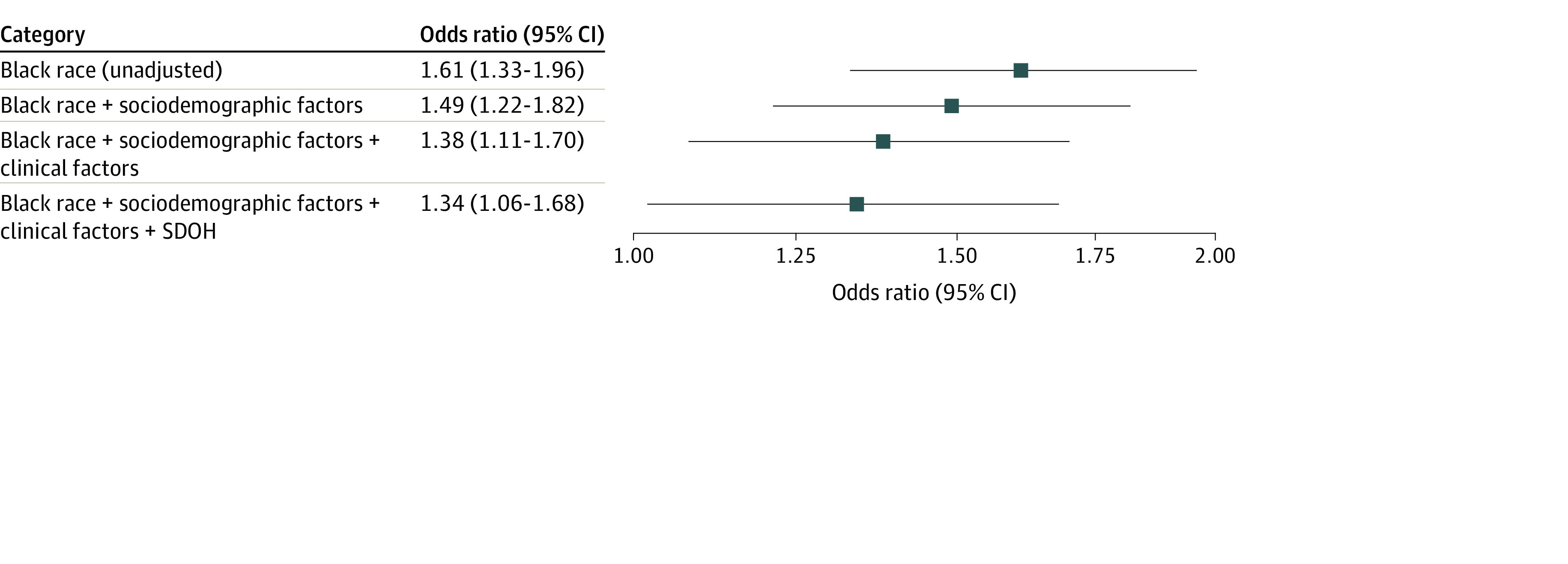
Association Between Self-reported Race and All-Cause 1-Year Hospital Readmission After Hospitalization for Acute Myocardial Infarction in Younger Adults SDOH indicates social determinant of health.

Specifically, Black individuals who were unemployed and those who had fewer work hours per week were more likely to be readmitted at 1 year. Conversely, Black individuals who were employed or with more work hours per week and White individuals in general were less likely to be readmitted. Subgroup analyses by employment status are presented in eTable 5 in Supplement 1. In all models, the interaction between race and sex was nonsignificant (model 1: OR, 0.90; 95% CI, 0.54-1.48; *P* = .67; model 2: OR, 0.92; 95% CI, 0.55-1.54; *P* = .74; model 3: OR, 0.95; 95% CI, 0.56-1.62; *P* = .85). Additional baseline characteristics considered but not included in the multivariable model are given in eTable 6 in Supplement 1. Finally, the Blinder-Oaxaca 2-way decomposition indicated that only 21% of the difference in readmission for Black race was explained by the significant covariates in model 3. The 79% that remained unexplained is attributable to unmeasured variables, including potential discrimination.

## Discussion

This cohort study set out to assess the association of race and SDOHs on readmission within 1 year of discharge from the hospital for AMI. We demonstrated that in a younger population of AMI survivors, Black individuals were at higher risk for all-cause hospital readmission up to 1 year compared with White individuals. The observed racial associations were slightly attenuated after adjustment for age, sex, cardiac risk factors, comorbidities, disease severity, and SDOHs. Although there was no interaction between race and sex, Black individuals who were unemployed or were employed with fewer work hours experienced an even higher risk of readmission.

Our study extends the literature in several important ways. First, although prior studies^4,5,6^ have assessed the association between race and various AMI outcomes, our study provides the first available evidence, to our knowledge, of racial disparities in all-cause readmission up to 1 year among younger individuals who have survived an AMI. Consistent with prior studies using clinical trial data or administrative data,^4,5,6^ our study found a nearly 50% increase in hospital readmission in Black individuals compared with their White counterparts of the same age and sex. Second, we provide insights into race-related heterogeneity in clinical and nonclinical characteristics between Black and White AMI survivors. Collectively, these findings show that readmission at 1-year after AMI disproportionately affects Black individuals, and clinical and psychosocial factors do not explain most of this difference.

Prior studies^5,6,7,27^ suggest that racial disparities in AMI outcomes were largely explained by a greater burden of comorbidities and cardiac risk factors of Black individuals compared with White individuals. Although Black and White participants in our study were significantly different in almost all patient-level characteristics, adjusting for those risk factors did not significantly alter the observed difference. Comorbidities, such as diabetes, history of depression, kidney disease, prior AMI, and physical inactivity, remained important factors associated with 1-year readmission, emphasizing that these high-risk groups may benefit from closer follow-up and better prevention.^28,29^ However, traditional cardiac risk factors and comorbidities alone cannot fully explain the racial differences in AMI readmission in younger individuals. The association of race, as a social construct,^30^ directly with these factors^31,32^ and indirectly through SDOHs^10,32,33^ should also be considered. Our study shows higher odds of readmission at 1 year for participants with low socioeconomic status, low social support, and high stress. These SDOHs have been shown to be related to structural racism, which inequitably limits opportunities for racial and ethnic minority groups.^34^ Our study broadens the existing evidence by taking into consideration a wide spectrum of SDOHs under the World Health Organization conceptual framework^32^ and highlights that Black participants have a greater burden of nonclinical risk factors compared with White individuals. Despite this burden, the measured SDOHs and other significant covariates from model 3 explained only 21% of the higher odds of readmission among Black participants. Qualitative work on AMI survivorship has conceptualized the recovery process as a deeply personal, unique process associated with daily functioning, which may result in changes in SDOHs, such as employment status, psychological stress, and perceived social support.^33^ Our findings necessitate further research to explore other unmeasured factors that explain the racial differences in readmission at 1 year, including structural racism^35^ and implicit bias,^35,36^ as well as more focus on addressing these upstream influences.

Our study shows a disproportionate burden of morbidity among younger Black women. Although we did not observe a significant sex-race interaction, it appears that close to half of younger Black women had a hospital readmission up to 1 year after AMI. A previous study^6^ on all-aged patients with AMI treated with percutaneous coronary intervention and an adenosine diphosphate receptor inhibitor reported a total of 29.5% unplanned rehospitalizations, with Black women having the highest rate (44.1%). Our study found similar results (30.8% overall readmission rate, 42.1% for Black women) in AMI survivors 10 years younger, on average, than in this previous study, suggesting important contributory factors of these differences. Our findings call for a more in-depth understanding of how intersecting identities, such as race, ethnicity, and biological sex, come together to impact health outcomes,^37^ especially as they relate to cardiovascular health^38,39^ and AMI specifically.

Our study provides evidence of racial disparities in long-term outcomes among younger AMI survivors, which calls for more clinical attention paid to this high-risk group. Although factors contributing to the racial disparities in 1-year AMI readmission rates remain to be fully identified, SDOHs such as employment status were shown to worsen the association between race and readmission. As a result, finding root causes of unemployment could provide a means of reducing readmission at 1 year while helping to mitigate economic racial disparity.^40^ In addition, our study found that health insurance status was not a significant factor for readmission at 1 year, with near similar proportions of Black and White individuals having health insurance despite a significantly higher proportion of low socioeconomic status among Black individuals. This finding may be explained by unmeasured factors such as unemployment benefits and government-sponsored insurance policies like Medicaid that provide increased health insurance^41,42^ and potential reduction in the readmission rate^43^ among enrollees. This finding further highlights the association of SDOHs with 1-year readmission after AMI and the need to address these factors beyond the individual level, at the structural and policy levels.

### Limitations

Several limitations of this study merit discussion. First, given the observational nature of the study, residual confounding due to unmeasured characteristics that differ by race may bias the results and therefore preclude any assessment of causality. Further research is needed to identify important explanatory factors between the observed racial disparities in AMI outcomes so that additional targets for intervention can be pinpointed. Second, the study relies on self-identified racial categories and only includes Black and White races, with participants indicating 1 racial category. The next generation of research could extend to other racial and ethnic groups and multiracial individuals and leverage genetic and ancestral data to provide further insights, as self-reported race as used in this study is a social construct and has no biological meaning.^30^ Third, although the VIRGO study participants were recruited from hospitals with diverse geographic representation across the US, the data constitute a modest cohort of Black individuals, and the study was conducted primarily in academic hospitals, potentially further limiting its generalizability. Fourth, although used in prior research,^9,25^ the combination of SDOH measures from the World Health Organization has not been previously validated.

## Conclusions

In this cohort study of younger adults hospitalized for AMI, Black individuals had a higher risk of all-cause readmission 1 year after AMI than White individuals, and this observed disparity remained significant after adjusting for sociodemographic characteristics, cardiac risk factors, comorbidities, disease severity, and SDOHs. Being unemployed was associated with an increased risk of readmission, and Black unemployed individuals had an even higher rate of readmission. This finding suggests that addressing patients’ SDOH risk may reduce racial disparities in the prognoses of patients with AMI. The fact that 79% of this racial difference in likelihood of readmission remains unexplained suggests there are other unmeasured variables that need to be explored as a foundation for developing strategies to reduce racial disparities in readmission after an AMI.

## Notes

Supplement 1. **eTable 1.** Missing Values for Baseline Characteristics and SDOH Stratified by Race and All-Cause 1-Year Readmission Post AMI **eTable 2.** Fully Adjusted Models in Each Race Subgroup **eTable 3.** Univariate Regression Showing Factors Associated With All-Cause 1-Year Readmission in Young Adults With AMI **eTable 4.** Generalized Mixed Effects Model Showing Factors Associated With All-Cause 1-Year Readmission in Young Adults With AMI After Accounting for Site Effect **eTable 5.** Comparison of All-Cause 1-Year Readmission Between Black and White Individuals Stratified by Employment Status **eTable 6.** Additional Baseline Characteristics Considered but Not Included in the Multivariable Model

Supplement 2.
Data Sharing Statement
