# Supplementary material for: Association of Sociodemographic Characteristics With 1-Year Hospital Readmission Among Adults Aged 18 to 55 Years With Acute Myocardial Infarction
Source: JAMA Netw Open. 2023 Feb 14;6(2):e2255843. doi: 10.1001/jamanetworkopen.2022.55843 (PMC9929697; doi:10.1001/jamanetworkopen.2022.55843)
Supplement: Supplement 1. — eTable 1. Missing Values for Baseline Characteristics and SDOH Stratified by Race and All-Cause 1-Year Readmission Post AMI eTable 2. Fully Adjusted Models in Each Race Subgroup eTable 3. Univariate Regression Showing Factors Associated With All-Cause 1-Year Readmission in Young Adults With AMI eTable 4. Generalized Mixed Effects Model Showing Factors Associated With All-Cause 1-Year Readmission in Young Adults With AMI After Accounting for Site Effect eTable 5. Comparison of All-Cause 1-Year Readmission Between Black and White Individuals Stratified by Employment Status eTable 6. Additional Baseline Characteristics Considered but Not Included in the Multivariable Model [file jamanetwopen-e2255843-s001.pdf]

Supplementary Online Content

Okafor CM, Zhu C, Raparelli V, et al. Association of sociodemographic characteristics with 1-year hospital readmission among adults aged 18 to 55 years with acute myocardial infarction. *JAMA Netw Open*. 2023;6(2):e2255843. doi:10.1001/jamanetworkopen.2022.55843

eTable 1. Missing Values for Baseline Characteristics and SDOH Stratified by Race and All-Cause 1-Year Readmission Post AMI

eTable 2. Fully Adjusted Models in Each Race Subgroup

eTable 3. Univariate Regression Showing Factors Associated With All-Cause 1-Year Readmission in Young Adults With AMI

eTable 4. Generalized Mixed Effects Model Showing Factors Associated With All-Cause 1-Year Readmission in Young Adults With AMI After Accounting for Site Effect

eTable 5. Comparison of All-Cause 1-Year Readmission Between Black and White Individuals Stratified by Employment Status

eTable 6. Additional Baseline Characteristics Considered but Not Included in the Multivariable Model

This supplementary material has been provided by the authors to give readers additional information about their work.

© 2023 Okafor CM et al. *JAMA Network Open.*

eTable 1. Missing Values for Baseline Characteristics and SDOH stratified by race and All-Cause 1-year readmission post-AMI.

**Variable**	**Total Missing (N, %)**	**Readmitted Missing**		**Not Readmitted Missing**	
		**Black**	**White**	**Black**	**White**
**Demographics (%)**					
Age	0 (0)	0 (0)	0 (0)	0 (0)	0 (0)
Sex	0 (0)	0 (0)	0 (0)	0 (0)	0 (0)
**Cardiac risk factors (%)**					
Diabetes	0 (0)	0 (0)	0 (0)	0 (0)	0 (0)
Obesity (BMI≥30 kg/m^2)	2 (0.1)	0 (0)	0 (0)	0 (0)	2 (0.1)
Hypertension	0 (0)	0 (0)	0 (0)	0 (0)	0 (0)
Dyslipidemia	0 (0)	0 (0)	0 (0)	0 (0)	0 (0)
Current Smoking	0 (0)	0 (0)	0 (0)	0 (0)	0 (0)
Family History of CVD	7 (0.3)	0 (0)	0 (0)	1 (0.3)	6 (0.4)
Inactivity	0 (0)	0 (0)	0 (0)	0 (0)	0 (0)
**Comorbidities/Medical history (%)**					
Prior MI	0 (0)	0 (0)	0 (0)	0 (0)	0 (0)
History of renal disease	10 (0.4)	2 (1.0)	1 (0.2)	1 (0.3)	6 (0.4)
Alcohol abuse	0 (0)	0 (0)	0 (0)	0 (0)	0 (0)
History of COPD	0 (0)	0 (0)	0 (0)	0 (0)	0 (0)
History of stroke	13 (0.5)	0 (0)	6 (0.9)	0 (0)	7 (0.4)
History of heart failure	0 (0)	0 (0)	0 (0)	0 (0)	0 (0)
History of PAD	13 (0.5)	1 (0.5)	3 (0.5)	2 (0.6)	7 (0.4)
History of recreational drug use	0 (0)	0 (0)	0 (0)	0 (0)	0 (0)
History of depression	1 (0.04)	0 (0)	0 (0)	0 (0)	1 (0.1)
**Disease severity (%)**					
Ejection Fraction >40%	85 (3.0)	8 (3.8)	21 (3.2)	10 (3.1)	46 (2.8)
AMI type	0 (0)	0 (0)	0 (0)	0 (0)	0 (0)
Admitted to CCU/ICU	119 (4.2)	8 (3.1)	31 (4.7)	20 (6.2)	60 (3.7)
**Social determinants of health**					
Low SES	91 (3.2)	10 (4.8)	16 (2.4)	15 (4.6)	50 (3.1)
Currently unemployed	0 (0)	0 (0)	0 (0)	0 (0)	0 (0)
No. of work hours per week	23 (0.8)	1 (0.5)	4 (0.6)	1 (0.3)	17 (1.0)
Married or living with spouse	0 (0)	0 (0)	0 (0)	0 (0)	0 (0)
Primary earner	0 (0)	0 (0)	0 (0)	0 (0)	0 (0)
High burden of stress	31 (1.1)	8 (3.8)	6 (1.0)	4 (1.2)	13 (0.8)
Support for household chores	32 (1.1)	7 (3.3)	8 (1.2)	7 (2.2)	10 (0.6)
Low social support	44 (1.6)	9 (4.3)	11 (1.7)	8 (2.5)	16 (1.0)
Has health insurance	8 (0.3)	0 (0)	2 (0.3)	0 (0)	6 (0.4)

BMI = Body mass index, CVD= Cardiovascular disease, COPD=Chronic obstructive pulmonary disease, PAD= Peripheral artery disease, SES=socioeconomic status

© 2023 Okafor CM et al. *JAMA Network Open.*

eTable 2. Fully adjusted models in each race subgroup.

	**Black (n=533)**	**White (n=2289)**
**Variable**	**Odds Ratio (95% Confidence Interval)**	**Odds Ratio (95% Confidence Interval)**
**Demographics**		
Female Sex	1.44 (0.82, 2.51)	1.29 (1.03, 1.62) ‡
Age	0.98 (0.95, 1.02)	0.98 (0.97, 1) ‡
**Cardiac risk factors**		
Diabetes	1.75 (1.14, 2.68) ‡	1.39 (1.13, 1.72) ‡
Hypertension	1.3 (0.72, 2.32)	1.07 (0.85, 1.33)
Obesity	0.93 (0.6, 1.45)	0.9 (0.73, 1.1)
Dyslipidemia	1.71 (0.93, 3.14)	0.98 (0.72, 1.32)
Current Smoking	1.2 (0.77, 1.87)	1.05 (0.84, 1.32)
Inactivity	1.5 (1, 2.24) ‡	1.22 (0.99, 1.49)
**Comorbidities/Medical history**		
Prior MI	1.14 (0.72, 1.81)	1.45 (1.14, 1.84) ‡
History of renal disease	2.5 (1.39, 4.48) ‡	1.07 (0.79, 1.46)
History of depression	1 (0.64, 1.58)	1.4 (1.14, 1.72) ‡
**Disease Severity**		
AMI type	0.8 (0.53, 1.21)	1.14 (0.93, 1.38)
**SDOH**		
Low SES	0.62 (0.38, 1)	1.31 (1.03, 1.65) ‡
Currently unemployed	1.12 (0.44, 2.78)	1.04 (0.67, 1.61)
Incremental reduction in work hours per week	1.02 (1.00, 1.04)	1 (0.99, 1.01)
Married or living with spouse	0.97 (0.6, 1.55)	1.07 (0.84, 1.35)
Primary earner	0.87 (0.54, 1.4)	0.82 (0.63, 1.06)
High burden of stress	1.4 (0.91, 2.16)	1.25 (1.02, 1.53) ‡
Low social support	1.3 (0.81, 2.1)	1 (0.78, 1.27)

‡ Statistically significant in model.

© 2023 Okafor CM et al. *JAMA Network Open.*

eTable 3. Univariate Regression Showing Factors Associated with All-Cause 1-Year Readmission in Young Adults with AMI.

**Variable**	**Univariate Analysis**	
	**Odds Ratio (95% Confidence Interval)**	**P-value**
**Socio-demographics**		
Black race (vs White race)	1.61 (1.32, 1.96)	<0.001
Age	0.99 (0.98, 1.00)	0.070
Female sex (vs Male sex)	1.71 (1.43, 2.05)	<0.001
**Cardiac risk factors**		
Diabetes	1.78 (1.51, 2.10)	<0.001
Obesity	1.17 (0.99, 1.37)	0.061
Hypertension	1.52 (1.27, 1.81)	<0.001
Dyslipidemia	1.25 (0.98, 1.60)	0.068
Current Smoking	0.91 (0.76, 1.09)	0.319
Family History of CVD	1.14 (0.96, 1.35)	0.146
Inactivity	1.48 (1.25, 1.74)	<0.001
**Comorbidities/Medical history**		
Prior MI	1.80 (1.49, 2.17)	<0.001
History of renal disease	1.55 (1.22, 1.96)	<0.001
Alcohol abuse	0.75 (0.63, 0.89)	0.001
History of COPD	1.85 (1.46, 2.34)	<0.001
History of stroke	1.88 (1.24, 2.83)	0.003
History of heart failure	3.21 (2.25, 4.58)	<0.001
History of PAD	3.02 (1.86, 4.89)	<0.001
History of recreational drug use	1.90 (1.04, 3.45)	0.036
History of depression	1.67 (1.42, 1.96)	<0.001
**Presentation Characteristics / Disease severity**		
Ejection Fraction >40%	1.27 (0.99, 1.63)	0.063
NSTEMI (vs STEMI)	1.26 (1.07, 1.47)	0.006
Admitted to CCU/ICU	1.10 (0.75, 1.63)	0.627
**Social Determinants of Health (SDOH)**		
Low SES	1.76 (1.49, 2.07)	<0.001
Unemployed Participants	1.87 (1.59, 2.20)	<0.001
Incremental reduction in work hours per week	1.01 (1.01, 1.02)	<0.001
Married or living with spouse	0.71 (0.60, 0.83)	<0.001
Primary earner	0.74 (0.62, 0.88)	<0.001
High burden of stress	1.48 (1.25, 1.73)	<0.001
Support for household chores	0.87 (0.73, 1.02)	0.092
Low social support	1.23 (1.02, 1.50)	0.032
Has health insurance	1.07 (0.88, 1.30)	0.482

BMI = Body mass index, CVD= Cardiovascular disease, COPD=Chronic obstructive pulmonary disease, PAD= Peripheral artery disease, STEMI=ST elevation myocardial infarction, NSTEMI= non-ST elevation myocardial infarction, CCU= critical care unit, ICU= intensive care unit

© 2023 Okafor CM et al. *JAMA Network Open.*

eTable 4. Generalized Mixed Effects Model Showing Factors Associated with All-Cause 1-year readmission in Young Adults with AMI after accounting for site effect.

	Model 1	Model 2	Model 3
**Variable**	**Odds Ratio (95% Confidence Interval)**	**Odds Ratio (95% Confidence Interval)**	**Odds Ratio (95% Confidence Interval)**
**Demographics**			
Self-reported Black Race	1.49 (1.17, 1.90)*	1.41 (1.10, 1.82)†	1.40 (1.07, 1.84)‡
Female Sex	1.67 (1.39, 2.00)*	1.48 (1.21, 1.81)†	1.33 (1.06, 1.63)‡
Age	0.99 (0.98, 1.00)	0.98 (0.96, 0.99)†	0.98 (0.97, 1.00)‡
**Cardiac risk factors**			
Diabetes		1.47 (1.21, 1.78)†	1.47 (1.20, 1.80)‡
Hypertension		1.14 (0.93, 1.41)	1.10 (0.89, 1.37)
Obesity		0.90 (0.74, 1.08)	0.86 (0.71, 1.05)
Dyslipidemia		1.08 (0.82, 1.42)	1.06 (0.80, 1.42)
Current Smoking		1.04 (0.85, 1.27)	1.16 (0.94, 1.44)
Inactivity		1.28 (1.06, 1.54)†	1.25 (1.03, 1.52)‡
**Comorbidities/Medical history**			
Prior MI		1.56 (1.26, 1.93)†	1.47 (1.17, 1.84)‡
History of renal disease		1.38 (1.06, 1.81)†	1.39 (1.05, 1.85)
History of depression		1.38 (1.15, 1.66)†	1.24 (1.02, 1.52)‡
**Disease Severity**			
AMI type		1.06 (0.88, 1.26)	1.04 (0.87, 1.26)
**SDOH**			
Low SES			1.10 (0.88, 1.38)
Currently unemployed			1.09 (0.72, 1.64)
Incremental reduction in work hours per week			1.01 (1.00, 1.02)
Married or living with spouse			1.00 (0.80, 1.25)
Primary earner			0.79 (0.62, 1.00)
High burden of stress			1.29 (1.06, 1.56)‡
Low social support			1.07 (0.85, 1.34)

Race/Sex interaction was also tested in each model and was not significant.

* Statistically significant in first model.

† Statistically significant in second model.

‡ Statistically significant in final model.

These results were obtained using the least-squared dummy variables (LSDB) model in R (Version 1.4)

© 2023 Okafor CM et al. *JAMA Network Open.*

eTable 5. Comparison of All-Cause 1-Year Readmission between Black and White individuals Stratified by Employment Status.

Variable	Unemployed Individuals			Employed Individuals		
	Black (N=266)	White (N=831)	P-value	Black (N=267)	White (N=1458)	P-value
**Readmitted**	130 (48.9)	298 (35.9)	<0.001	80 (30.0)	360 (24.7)	<0.001
**Not Readmitted**	136 (51.1)	533 (64.1)		187 (70.0)	1098 (75.3)	

eTable 6. Additional Baseline Characteristics Considered but Not Included in the Multivariable Model.

Variable	Total (N=2822)	Readmitted (N = 868)		P-value*	Not Readmitted (N=1954)		P-value*
		Black (N= 210)	White (N= 658)		Black (N= 323)	White (N= 1631)	
**Presentation Characteristics / Disease severity (%)**							
First health service used				<0.001			0.009
Direct to ER from home	2513 (89.1)	195 (92.9)	574 (87.2)		302 (93.5)	1442 (88.4)	
Before ER, Dr Office	155 (5.5)	12 (5.7)	42 (6.4)		14 (4.3)	87 (5.3)	
Before ER, other health services	154 (5.5)	3 (1.4)	42 (6.4)		7 (0.3)	102 (6.3)	
Late Presentation >6h	1245 (44.3)	99 (47.4)	304 (46.3)	0.796	174 (54.2)	668 (41.1)	<0.001
Acetylsalicylic acid at arrival	2707 (97.7)	197 (97.0)	626 (97.4)	0.812	309 (97.5)	1575 (98.0)	0.595
Obstructive CAD ≥50%	2287 (90.5)	168 (91.3)	538 (92.3)	0.468	247 (83.5)	1334 (90.9)	<0.001
Peak Troponin (Mean ± SD)	26.4 ± 54.96	19.7 ± 48.5	27.2 ± 57.7	0.023	19.9 ± 42.6	28.2 ± 56.7	<0.001
Estimated Glomerular Filtration Rate (eGFR)	88.3 ± 24.3	88.2 ± 33.9	85.8 ± 24.5	0.150	96.9 ± 27.4	87.7 ± 21.6	<0.001
First White Blood Cell Count	10.8 ± 3.9	9.3 ± 3.2	11.1 ± 4.1	<0.001	9.2 ± 3.2	11.2 ± 3.9	<0.001
First Hematocrit	41.0 ± 5.2	37.7 ± 5.7	41.0 ± 5.6	<0.001	38.9 ± 5.1	41.9 ± 4.8	<0.001
Chest pain as primary symptom	2470 (87.5)	185 (88.1)	556 (84.5)	0.199	294 (91.0)	1435 (88.0)	0.118
Killip class				0.008			0.006
I (no rales)	2565 (95.8)	187 (94.0)	586 (93.5)		294 (97.0)	1498 (96.8)	
II (rales in bases / S3)	80 (3.0)	7 (3.5)	30 (4.8)		6 (2.0)	37 (2.4)	
III (rales over ½ the lungs / Pulmonary edema)	20 (0.8)	4 (2.0)	7 (1.1)		3 (1.0)	6 (0.4)	
IV (Cardiogenic shock)	12 (0.5)	1 (0.5)	4 (0.6)		0 (0.0)	7 (0.5)	
Prior coronary artery bypass grafting (CABG)	111 (4.0)	10 (4.8)	45 (6.9)	0.284	11 (3.4)	45 (2.8)	0.528
Global Registry of Acute Coronary Events Score (Mean ± SD)	75.1 ± 19.0	76.7 ± 24.3	76.1 ± 20.1	0.977	74.0 ± 20.0	74.7 ± 17.6	0.247
Conservative treatment	81 (2.9)	10 (4.8)	27 (4.1)	0.681	7 (2.2)	37 (2.3)	0.911

© 2023 Okafor CM et al. *JAMA Network Open.*

Total length of stay in days, (Mean ± SD)	4.19 ± 3.8	4.9 ± 3.6	4.9 ± 4.8	0.076	3.9 ±3.4	3.9 ± 3.4	0.487
Disposition to other institutions at discharge	26 (1.0)	2 (1.0)	12 (1.9)	0.360	2 (0.6)	10 (0.7)	0.982
**Discharge Counseling (%)**							
Recommended Counselling (Cardiac+Diet+Smoking)	914 (32.4)	53 (25.2)	214 (32.5)	0.046	76 (23.5)	571 (35.0)	<0.001
Medication Counselling	2781 (98.6)	207 (98.6)	646 (98.2)	0.702	319 (98.8)	1609 (98.7)	0.874
Exercise Counselling	2605 (92.3)	196 (93.3)	610 (92.7)	0.758	286 (88.5)	1513 (92.8)	0.010
Clopidogrel (Thienopyridines)	1955 (69.3)	133 (63.3)	452 (68.7)	0.149	218 (67.5)	1152 (70.6)	0.260
Statins	2602 (92.2)	197 (93.8)	604 (91.8)	0.341	298 (92.3)	1503 (92.2)	0.947
Dual Antiplatelet Therapy	1872 (66.3)	128 (61.0)	424 (64.4)	0.361	206 (63.8)	1114 (68.3)	0.113
ACEi/ARBs	1822 (64.6)	144 (68.6)	417 (63.4)	0.170	212 (65.6)	1049 (64.3)	0.651
Beta Blockers	2573 (91.2)	190 (90.5)	596 (90.6)	0.965	286 (88.5)	1501 (92.0)	0.041
Calcium Channel Blocker	141 (5.0)	21 (10.0)	29 (4.4)	0.003	26 (8.1)	65 (4.0)	0.002
**In-hospital complications (%)**							
Bleeding	191 (6.8)	10 (4.8)	53 (8.1)	0.109	17 (5.3)	111 (6.8)	0.306
Re-infarction	27 (1.0)	5 (2.4)	8 (1.2)	0.225	3 (0.9)	11 (0.7)	0.623
Heart failure	200 (7.2)	21 (10.1)	73 (11.3)	0.644	20 (6.3)	86 (5.3)	0.495
Cardiac arrhythmias	187 (6.7)	11 (5.3)	58 (8.9)	0.093	8 (2.5)	110 (6.8)	0.003
**Psychosocial factors** (Mean ± SD) (%)							
Social Support (ESSI-5)	21.3 ± 4.6	20.3 ± 5.6	21.1 ± 4.9	0.349	21.4 ± 4.4	21.5 ± 4.4	0.989
Depression (PHQ-9)	7.9 ± 6.5	9.0 ± 6.7	9.6 ± 6.7	0.254	7.1 ± 5.9	7.2 ± 6.3	0.992
Stress (PSS-14)	26.1 ± 9.8	26.8 ± 9.8	28.0 ± 9.3	0.203	25.3 ± 8.9	25.4 ± 10.1	0.935
Physical Limitations (SAQ)	80.3 ± 25.9	67.6 ± 30.4	75.4 ± 28.2	<0.001	75.5 ±28.2	84.8 ± 22.7	<0.001
Angina Frequency (SAQ)	83.0 ± 20.9	75.1 ± 26.6	80.5 ± 22.7	0.014	82.3 ± 22.0	85.1 ± 18.6	0.183
Treatment satisfaction (SAQ)	91.7 ± 13.0	88.4 ± 16.9	90.7 ± 14.1	0.355	90.4 ± 14.2	92.9 ± 11.5	0.034
Quality of life (SAQ)	57.3 ± 25.1	49.6 ± 26.4	53.1 ± 25.3	0.115	57.2 ± 25.4	59.9 ± 24.3	0.074
General health, SF-12 (PCS)	42.8 ± 12.1	37.5 ± 12.7	39.7 ± 12.5	0.042	42.0 ± 11.5	44.9 ± 11.6	<0.001
General health, SF-12 (MCS)	45.4 ± 12.4	44.4 ± 12.9	43.5 ± 12.9	0.537	46.2 ± 12.0	46.1 ± 12.1	0.992

* P-values for comparison of Black and White race within strata defined by readmission status from Kruskal-Wallis and chi-square tests respectively for continuous and categorical variables

© 2023 Okafor CM et al. *JAMA Network Open.*
