# Supplementary material for: Association of Sociodemographic Characteristics With 1-Year Hospital Readmission Among Adults Aged 18 to 55 Years With Acute Myocardial Infarction
Source: JAMA Netw Open. 2023 Feb 14;6(2):e2255843. doi: 10.1001/jamanetworkopen.2022.55843 (PMC9929697; doi:10.1001/jamanetworkopen.2022.55843)
Supplement: Supplement 2. — Data Sharing Statement [file jamanetwopen-e2255843-s002.pdf]

Data Sharing Statement

Okafor. Association of Sociodemographic Characteristics With 1-Year Hospital Readmission Among Adults Aged 18 to 55 Years With Acute Myocardial Infarction. *JAMA Netw Open*. Published February 14, 2023. doi:10.1001/jamanetworkopen.2022.55843

Data
Data available: No

Additional Information
Explanation for why data not available: All relevant data are within the manuscript and its Supporting Information files. We are open to outside investigators obtaining access to the full VIRGO data and may contact us at y.lu@yale.edu (Yuan Lu is the principal investigator of the VIRGO study).
